# FINANCIAL IMPLICATIONS OF DEMENTIA: AN EIGHT-YEAR FOLLOW-UP OF MATCHED SAMPLES

**DOI:** 10.1093/geroni/igae098.1274

**Published:** 2024-12-31

**Authors:** HwaJung Choi, Kenneth Langa, Edward Norton, Tsai-Chin Cho, Cathleen Connell

**Affiliations:** University of Michigan, Ann Arbor, Michigan, United States; University of Michigan, Ann Arbor, Michigan, United States; University of Michigan, Ann Arbor, Michigan, United States; University of Michigan, Ann Arbor, Michigan, United States; University of Michigan, Ann Arbor, Michigan, United States

## Abstract

Financial consequences of dementia could be devastating because of the extensive care needs and high care costs associated with dementia. This study is to determine the financial status of older adults before and after the onset of dementia and assess the potential implications for individuals, families, and socieity. This population-based cohort study compared two matched samples of U.S. adults aged 55 years or older from national, longitudinal data. To create matched samples (dementia vs. control), the study included extensive baseline variables of sociodemographic characteristics, economic status, family availability, health conditions, disability status, and outpatient care use. In total, 2387 adults experienced the onset of dementia during the 2-year follow-up (dementia group) and 2387 adults did not (control group). Participants were followed up for 8 years from the baseline. Over the 8-year follow-up in the dementia group, the 2-year out-of-pocket medical costs increased from $4005 to $ 10,006, median wealth was reduced from $79,339 to $30,490, and those enrolling in Medicaid increased from 16.1% to 29.7%. No statistically significant changes in financial outcomes were found in the control group. The study found that in-home medical care, hospital stay, and nursing home facility stay increased dramatically, especially within two years after the onset of dementia. Greater family care availability is significantly associated with a lower level of transition to a nursing home, which is the most expensive care setting. The potential mitigating effect of family care availability should be accounted for in developing an intervention to reduce the financial effects of dementia.

